# Establishment of a spontaneous ventilation anesthesia model for beagle dogs

**DOI:** 10.1016/j.mex.2024.102607

**Published:** 2024-02-06

**Authors:** Yuan Zeng, Hui Liu, Fei Cui, Weipeng Cai, Jianxing He, Jun Liu

**Affiliations:** a Department of Thoracic Surgery & Department of Organ Transplantation, The First Affiliated Hospital of Guangzhou Medical University, Guangzhou, China; b Guangzhou Institute of Respiratory Disease & China State Key Laboratory of Respiratory Disease & National Clinical Research Center for Respiratory Disease, Guangzhou, China; c Department of Anesthesia, The First Affiliated Hospital of Guangzhou Medical University, Guangzhou, China; d Department of Cardiothoracic Surgery, Shantou Central Hospital, Shantou, China

**Keywords:** Spontaneous ventilation, Beagle dog, Anesthesia model, Spontaneous ventilation anesthesia model for beagle dogs

## Abstract

While spontaneous ventilation (SV) anesthesia is in use for clinical patients, there is still little systematic experimental research into its basic aspects. The rabbit SV model that we established previously has some limitations including the model being too small, differences in anesthetic drugs and anesthesia procedures, so we set out to establish an SV anesthesia model for beagle dogs.

• Single lumen tracheal intubation was performed on beagles connecting a ventilator, and the anesthetic dosage was adjusted for spontaneous ventilation before surgery.
• 5 mL of 1 % lidocaine was applied as a local infiltration anesthesia at the surgical incision.
• After thoracotomy, 5 mL of 1% lidocaine was sprayed onto the surface of the lungs and a T3–T7 intercostal nerve block (1:1 2 % lidocaine:0.75 % ropivacaine) was performed.

## Graphical abstract

The flowchart of establishing a spontaneous ventilation anesthesia model for beagle dogs.

**Figure:**
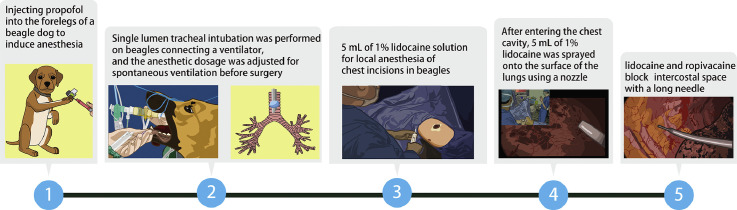

Specifications table

**Table:** 

Subject area:	Medicine
More specific subject area:	Thoracic Surgery, Anesthesia
Name of your method:	Spontaneous ventilation anesthesia model for beagle dogs
Name and reference of original method:	Chen, Z.; Dong, Q.; Liang, L. Effect of different thoracic anesthesia on postoperative cough. J Thorac Dis. 2018, 10, 3539–3547.
Resource availability:	NA

**Methods details**

## Background

Conventionally, video-assisted thoracoscopic surgery (VATS) is carried out under general anesthesia with the insertion of a double-lumen endotracheal tube and one-lung ventilation (OLV) [1]. Characteristically, spontaneous ventilation (SV) VATS does not use muscle relaxants to maintain the patient in a state of sedation and analgesia, and allows the patient to breath spontaneously without mechanical ventilation [2]. At present, SV anesthetic technology has been applied to various thoracoscopic surgeries [3], [4], [5]. However, SV anesthesia for thoracic surgery is not in wide use globally, and controversy still exists regarding its safety and whether it is superior to traditional thoracic surgical anesthesia. Moreover, there is currently little systematic basic experimental research into SV. We have previously simulated thoracic surgery using rabbits and found that lung injury may be more severe in those subjected to SV where the operation time exceeds 4 h [6]. However, this rabbit model has some limitations due to its small size, differences in anesthetic drugs and anesthesia procedures. Therefore, we used a larger animal (beagle dogs) to establish an improved SV anesthesia model and to simulate typical clinical conditions as closely as possible.

## Methodology

The healthy beagles, weighing 12.0 ± 1 kg were fasted and deprived of water from 10 p.m. the day before the experiment. The experiment was started approximately 10 h later. A tube was placed into the radial vein of the left forelimb through which 3–4 mg/kg of propofol was injected. After sedation, the animals were placed in the supine position and secured to a conventional operating table for large animals. The limbs and maxillofacial region were secured with elastic bandages and the skin was then prepared. According to the animal's reaction, 2–3 mg/kg of propofol was added as needed. The dogs were then endotracheally intubated and connected to an ECG monitor. A No. 7 single-lumen endotracheal tube was used for endotracheal intubation, and an electronic fiber bronchoscope was used to confirm that the intubation was well-positioned in the main bronchus. We secured the endotracheal intubation in a suitable position with a bandage, connected the ventilator circuit, and used the synchronized intermittent mandatory ventilation mode for auxiliary ventilation (tidal volume: 12 mL/kg, respiratory rate: 20 times/min, respiratory ratio: 1:1.5, oxygen concentration: 100 %). A left femoral artery puncture was performed, a No. 20 single-lumen central venous catheter was inserted, and a pressure transducer was connected. The invasive arterial blood pressure was measured. The right external jugular vein was punctured and a No. 20 single-lumen central venous catheter was inserted for infusion.

This SV anesthesia model presents improvements based on the current clinical anesthetic methods used in our hospital [7]. The maintenance medication contained: propofol 2–5 mg/kg/h, remifentanil: 0.05–0.1 µg/kg/min, epinephrine: 0.1–0.2 µg/kg/min, and norepinephrine: 0.1–0.3 µg/kg/min. Appropriate adjustments were made according to the reaction of the dogs to the anesthesia. In addition, depth of sedation and analgesia was monitored by an anesthesiologist. If necessary, flurbiprofen (10 mg) was given for analgesia. After the dogs’ breathing rate and blood oxygen levels had stabilized, we stopped ventilator-assisted breathing before opening the chest and switched to the autonomous breathing mode. At this point, the beagle is still connected to the ventilator and the condition of airway resistance is continuously monitored. During the operation, we performed a local intercostal block using 5 mL of 1 % lidocaine stock solution, a right thoracotomy with a 2.5 cm incision on the fifth intercostal, inserted a protective sleeve at the incision, sprayed 5 mL of 1 % lidocaine stock solution on the lung surface, and then performed an intercostal nerve block using a 1:1 mixture of 2 % lidocaine:0.75 % ropivacaine to block the 3rd, 4th, 5th, 6th, and 7th intercostal nerves. Each intercostal nerve block was performed using 1 mL of drug solution. Ropivacaine in intercostal blockade can provide sustained pain relief for 4–6 h.

## Funding

Guangdong Provincial Basic and Applied Basic Research Fund of China (Grant No. 2020A1515110445); The China State Key Laboratory of Respiratory Disease Independent Subject (Grant No. SKLRD-QN-201925);

## Ethical statement

The study was approved by Ethics committee of First Affiliated Hospital of Guangzhou Medical University (approved NO. 2020166; Issued date 7/8/2020).

## Declaration of competing interest

The authors declare that they have no known competing financial interests or personal relationships that could have appeared to influence the work reported in this paper.

## Data Availability

Data will be made available on request.
